# The Best Predictor of the Future—the Metaverse, Mental Health, and Lessons Learned From Current Technologies

**DOI:** 10.2196/40410

**Published:** 2022-10-28

**Authors:** David Benrimoh, Forum D Chheda, Howard C Margolese

**Affiliations:** 1 Department of Psychiatry McGill University Montreal, QC Canada; 2 McGill University Healthcare Center Montreal, QC Canada

**Keywords:** metaverse, mental health, social media, virtual reality, VR, digital experience, human interaction, mental health risk, teleworking, assisted therapy, teletherapy, benefits, safety, mental health problems, data security, privacy, protection, user safety, safety regulations, mobile phone

## Abstract

The metaverse—a virtual world accessed via virtual reality technology—has been heralded as the next key digital experience. It is meant to provide the next evolution of human interaction after social media and telework. However, in the context of the growing awareness of the risks to mental health posed by current social media technologies, there is a great deal of uncertainty as to the potential effects of this new technology on mental health. This uncertainty is compounded by a lack of clarity regarding what form the metaverse will ultimately take and how widespread its application will be. Despite this, given the nascent state of the metaverse, there is an opportunity to plan the research and regulatory approaches needed to understand it and promote its positive effects while protecting vulnerable groups. In this viewpoint, we examine the following three current technologies whose functions comprise a portion of what the metaverse seeks to accomplish: teleworking, virtual reality, and social media. We attempted to understand in what ways the metaverse may have similar benefits and pitfalls to these technologies but also how it may fundamentally differ from them. These differences suggest potential research questions to be addressed in future work. We found that current technologies have enabled tools such as virtual reality–assisted therapy, avatar therapy, and teletherapy, which have had positive effects on mental health care, and that the metaverse may provide meaningful improvements to these tools. However, given its similarities to social media and its expansion upon the social media experience, the metaverse raises some of the same concerns that we have with social media, such as the possible exacerbation of certain mental health problems. These concerns led us to consider questions such as how the users will be protected and what regulatory mechanisms will be put in place to ensure user safety. Although clear answers to these questions are challenging in this early phase of metaverse research, in this viewpoint, we use the context provided by comparator technologies to provide recommendations to maximize the potential benefits and limit the putative harms of the metaverse. We hope that this paper encourages discussions among researchers and policy makers.

## Introduction

It is 8 AM on Tuesday, and you are in your therapist’s office for your weekly session. You listen to your therapist while enjoying the calming sound of the small water fountain in her office. Suddenly, you remember—today is the important meeting with your boss at 9 AM. Your therapist’s office is an hour away from your workplace—there is no way to get there on time. You almost have a panic attack. Just as suddenly, you remember—you are not really in her office. Owing to the strikingly authentic look of the surroundings, you forgot that you were attending your weekly session in the *metaverse*. You finish the session at 8:50 AM, remove your virtual reality (VR) headgear, and walk around the house to stretch your legs. It is 8:55 AM. You put the headset back on, choose your office avatar, and enter the meeting 5 minutes early. You sigh and think, “Thank goodness for technology!”

The term “metaverse” was coined by writer Neal Stephenson in his 1992 science fiction novel, *Snow Crash*, in which characters used digital avatars of themselves as a way of escaping a dystopian reality. However, since then, the term has evolved to refer to a technology that encompasses more than just a digital escape. Today, the metaverse is a virtual world that exists beyond the physical world, equipped with means for the creation of digital locations for work, play, and socializing. This virtual world is accessed using VR hardware plugged into the next iteration of major social networks and collaboration software.

Despite the definition, the fact is that what *precisely* the metaverse will be and how it will evolve over time remain unknown. Experience has taught us that it is easy to both over- and underestimate the impacts of new technologies and often impossible to predict their diverse applications. Decoding the human genome has not led to the widespread adoption of gene therapy for most diseases; by contrast, the myriad uses of the internet likely go far beyond what its progenitors would have imagined. It is not even clear, despite the posturing of large companies in this space, whether the metaverse will become a truly pervasive phenomenon, such as the social media that precedes it, or whether it will become a niche experience, relevant in only some industries and consumer segments. The pace of development and deployment of the metaverse also remains unclear, and the question of who gains access first will certainly have potential effects in terms of what kinds of mental and physical health concerns the metaverse may be able to help alleviate or exacerbate. We also are unlikely to be able to draw meaningful and generalizable conclusions based on the data collected from current users of the nascent metaverse as this group is unlikely to be representative of the general population. However, it remains possible that the metaverse will be restricted, at least initially, to specific subpopulations; should this be the case, research may need to be focused on those populations to reduce harms and maximize benefits of the new technology.

Having acknowledged these uncertainties, in this viewpoint, we will adopt the following as our guiding question: “What will the effects of the metaverse be on mental health?” We use this frame to consider some initial questions that researchers can address. We begin with the following question while drawing on the existing literature on social media, teleworking, and VR technologies: “In what ways might the metaverse differ from existing technologies?” We selected these 3 technologies as comparators to inform this viewpoint given their relevance to the likely uses of the metaverse.

## Comparator Technologies

Our 3 chosen comparator technologies (telework, VR, and social media) exist at varying levels of adoption and maturity. Especially in the context of the COVID-19 pandemic, many people—including providers of mental health care—have become familiar with working from home and conversing with team members through technology. As such, teleworking technologies can be said to be at a more advanced stage of adoption and maturity in both the consumer and medical realms.

A subset of video gamers has adopted VR technologies, although the numbers are limited compared with traditional platforms owing to cost and concerns with motion sickness [1]. VR programs have been proven to be of value in treating some mental health conditions such as phobias and posttraumatic stress disorder [2,3], although a 2019 meta-analysis concluded that VR may be equivalent to active comparators for posttraumatic stress disorder (with the caveat that this was based on a limited number of trials focusing mostly on male military service members) [4]. VR has also recently been used to reduce agoraphobia in people with psychosis; this therapy was automated and required minimal intervention from staff, although staff with varying degrees of training were present in the room, helped review homework assigned during VR therapy, and encouraged patients to apply what they learned in the real world [5]. Although VR has seen some adoption in consumer and clinical contexts, its maturity and use are perhaps the lowest of the 3 technologies.

Social media has a ubiquitous influence on the lives of billions of people [6], but research on social media continues to present significant challenges, including data access and quality [7,8]. Despite these challenges, there is growing and consistent evidence of potential negative effects of social media use and misuse on mental health, particularly in children and adolescents. In a recent large study, higher amounts of social media use has been shown to predict later reductions in life satisfaction [9]. It is worth noting from recent reports that social media companies were aware of the negative impacts on the mental health of adolescents based on research conducted internally, including worsening body image issues, increased feelings of depression and anxiety, and suicidal ideation, but chose to keep these data private [10]. Despite these findings, it must be acknowledged that research on social media is still a rapidly changing field that is continuously providing new nuances to our understanding. For example, some studies have found that passive social media use (eg, monitoring social media feeds and passively consuming content by others) has been associated with worsened well-being, whereas active use (ie, using social media for the purpose of connecting with others or making posts) is associated with improved well-being; however, in a recent review, these findings were not shown to be consistent across studies, potentially as the benchmarks used to measure active versus passive use, such as time, were not precise enough [11]. The authors further discussed the possibility that future research needs to consider the profiles of users as well as the type of engagement in social media, highlighting the potential importance of taking a nuanced approach that accounts for both the technology and individual differences in this kind of research. Social media has the most advanced adoption and maturity from a consumer standpoint, although it currently seems to be rather limited with respect to both metrics in terms of clinical application. Research on each of these technologies has something to teach us about the metaverse’s potential effect on mental health. While exploring this, let us also clarify some potential distinctions between these technologies and the metaverse.

## Telework

In the case of telework, the purpose of relevant technologies (such as team management applications and videoconferencing) is to enable workers to continue working *from* home. The mental health effects of telework are known to be complex and depend on variables such as available organizational support, social connections available outside of work, and work-family conflict (see the review by Oakman et al [12] and the study by De Sio et al [13]). We argue that the metaverse is qualitatively different, as its effect would be to enable workers to *be* at work while they are at home. The psychological effect this will have is unclear, but one might imagine that being able to interact with colleagues in a VR space may reduce the isolation that some teleworkers experience [14] while at the same time further eroding the separation between work and home life and potentially increasing the work-family conflict, which can reduce well-being during telework. We believe that the potential impact on people with anxiety disorders, such as social anxiety, should also be considered. On the one hand, we might imagine that coming to work as an avatar whose physical reactions are limited and likely controlled by the user may reduce anxiety and facilitate workplace integration for people with anxiety who fear that they might show symptoms of panic or tension publicly. On the other hand, we (speaking as therapists and psychiatrists who treat anxiety) might argue that this may also facilitate avoidance, leading to therapy-interfering avoidance behaviors or fewer people seeking care as they are able to support themselves while remaining isolated. Indeed, avoiding exposure during anxiety treatment is a common therapy-interfering behavior that can hinder treatment outcomes and that therapists associate with worsened outcomes [15]. Furthermore, other aspects of telework, such as social isolation, a perceived lack of support, poor sleep, or the need to sort through technical difficulties, could all contribute to increasing anxiety and reducing well-being [16-18], and as such, it is difficult to know without further empirical work which aspects of telework in the metaverse may drive or reduce anxiety and how this might interact with individual differences. Indeed, in line with the discussions by Valkenburg et al [11,19] and Oakman et al [12], we argue that an understanding of individual differences in responses to the metaverse will be as key to its future study as it may be to the study of social media and telework. In addition, although research on working in the metaverse is in its nascent stages, a recent study did find reduced subjective productivity and well-being as well as physical side effects such as nausea and migraine after a week of office work in VR [20]. This is certainly preliminary work, and it is possible that advances in technology and software application design will reduce some of these effects, but it does indicate that the metaverse is far from ready to positively transform work at present.

## Teletherapy and VR

A particular aspect of telework that intersects with mental health is the provision of teletherapy. There is already evidence that internet and videoconferencing–based psychotherapy seems to be as effective as in-person therapy for a number of indications, such as anxiety and depression [21,22], although there remains a need for more rigorous research in this area, especially considering the reduced tendency to recruit patients who are more severely ill or suicidal in teletherapy studies [23]. The metaverse could allow for easier integration of VR elements into traditional therapy (imagine a teletherapy session that transitions to a VR environment for exposure work). However, the question remains as to whether the metaverse will present significant advantages over in-person or existing VR and teleconferencing technologies. Should the metaverse become widely used, it may increase access to VR and teletherapy, which would be a significant benefit but which, we argue, would not in and of itself change the therapeutic process. However, we can also imagine that the metaverse may also present entirely new therapeutic opportunities. If it becomes truly trivial to take a session from a traditional (though virtual) therapist’s office to a crowded street or a public speaking engagement, it may make the treatment of anxiety disorders, such as social anxiety or panic disorder, more effective (given the importance of exposure in these conditions and the aforementioned evidence of the value of VR in treating anxiety disorders) and reduce disparities in care quality for those in rural areas. By providing more virtual environments in which a patient and therapist may move around safely, we believe that a host of functional assessments, novel behavioral tests, and new approaches to the therapeutic process become possible. Indeed, a parallel can be seen in the extensive literature examining the use of data captured by smartphones to better diagnose and “digitally phenotype” patients (see the review by Huckvale et al [24] for a discussion). We foresee that challenges are likely to present themselves; for example, conducting therapy when both participants are “avatars” may reduce key nonverbal cues that are still present to some extent during teleconferences and that are traditionally considered to be an important part of therapy [25]. As such, we might argue that VR approaches that better approximate real movement and reproduce the participants’ faces with high fidelity may be necessary to make full use of this technology. However, these are technological challenges that could realistically be solved.

Teletherapy did not *improve* therapy; it improved its availability and the ease of engagement for those who otherwise would have avoided the therapy or been unable to access services. For the metaverse to provide anything more than an incremental improvement over teletherapy, we argue that therapists and patients will need to be given tools and the capacity to create and manipulate content in the metaverse. This can be thought of as being similar to the map editors one might find in popular video games, where players can create new levels or “maps” to play on and share them with the community. Researchers and commercial interests may also generate standardized testing environments or scenarios, similar to the aforementioned current VR simulations used for therapy, which therapists may choose to integrate into their practice.

A key point of VR therapy as it currently exists is that, as noted previously, it is generally an extension of existing therapeutic practices guided by trained staff. This suggests that for full benefit and potentially to avoid harm, professionally guided use of metaverse-enabled therapy may be necessary.

The metaverse may also provide a useful adjunct in other areas where technology has been used to try to improve mental health. Let us take avatar therapy as an example. In this novel therapy, which has been used in psychotic disorders and has shown promise in treating persistent verbal hallucinations, patients construct a virtual representation of the persecutor and then engage in a dialogue with this “avatar,” who is voiced by the therapist [26]. Access to this therapy might be facilitated by the metaverse given the ease with which avatars can be created in virtual worlds. The metaverse may also pose challenges specific to those with psychosis; however, a digital world in which movements are tracked, environments can be controlled, and reality can be altered on a whim may, speculatively, worsen paranoid feelings or further entrench delusional beliefs.

In addition, the metaverse may be a useful tool for measurement-based care, a gold standard in the treatment of conditions such as depression. Measurement-based care entails the use of frequent standardized assessments to guide treatment. Within the metaverse, clinical researchers may find new and relevant measurements derived from social interaction, work habits, and other behaviors that could serve as more naturalistic markers of function and illness or act as predictors of treatment response or guides for the modification of treatment. However, as will be discussed in the following sections, concerns regarding privacy and the manner in which the metaverse will be monetized will pose a challenge to this use.

## Social Media

Having considered VR, teletherapy, and telework, let us turn to social media. Despite the aforementioned dangers, social media is very popular because it meets the human need to connect and share, and it can have positive effects on human connections [27]. The metaverse will include, as a key element, the experience of social media in VR. This means that the same concerns we currently have regarding social media can be applied to the metaverse. This raises a number of questions. Can people become addicted to the metaverse as some have argued people can become addicted to social media [28]? Can it exacerbate underlying symptoms of eating disorders, anxiety, and depression in a manner similar to social media? Will it, similar to social media, lead to a reinforcement of maladaptive sleep or physical activity patterns in some users [29]? Will it provide even more opportunities for bullying and abuse, especially now that people will have (virtual) bodies available to attack in addition to their social media profiles? There has already been evidence of the potential for virtual sexual harassment. A number of people, from researchers to metaverse beta testers, have reported being groped, pinched, and sexually and verbally assaulted in the metaverse by perpetrators who feel especially emboldened by the anonymity it provides [30]. In addition, the development of haptic technology, where a user can feel the stimulus of virtual punches or kicks on their physical body, opens doors for users now being exposed to physical assault along with virtual assault. As discussed previously, the effects of social media are complex and likely depend a great deal on the way in which people use it and on individual differences between users, and social media clearly has significant utility for a large segment of the population. There is no reason to expect that this will be different for the metaverse; therefore, the focus on negative aspects in this section is not intended to paint all social media as negative but rather to demonstrate areas of concern that likely need to be carried forward into metaverse research. Indeed, in our view, the social elements of the metaverse, which become amenable to measurement and research in their digital form, may prove to be rich avenues for research into social interactions as markers of function or measures for measurement-based care, as described previously.

Aside from existing concerns, we must also consider in what way the metaverse will represent a qualitative evolution of current social media. To address this, it is necessary to speculate to a certain extent, given that the metaverse is not yet available for meaningful empirical testing. Currently, social media is accessed *through* an interface—a phone or computer. This has not stopped it from being a powerful force in the lives of many. However, we ask how this might change when the interface (the VR headset) provides access to a virtual world that the user *inhabits* and this world itself *is, to a large extent,* social media. How will our relationship with social media evolve when what we identify as our bodies is subjected to the pressures of this virtual world? Indeed, as discussed in the study by Fardouly and Vartanian [31], it has already been demonstrated that social media use, especially prolonged use, can lead to more negative body image. If someone can morph and control their avatar to fit in with expectations, what kind of dissonance will be created when they emerge into the real world and remember that their real bodies cannot be altered with the same ease? This perhaps is the qualitative difference between the metaverse and current social media—one may create a persona for social media, whereas, in the metaverse, one may be able to create a new *person*. What this will mean for conditions in which self-esteem and identity are already deeply affected—such as personality and eating disorders—is unclear, but we argue that what is clear is the need for careful and concerted research in this area. Indeed, there is research demonstrating that teenage girls craft their personas on the web and often hide feelings while on the internet and, furthermore, that the nature of the persona they craft depends on the social media site they use and the environment it creates [32], and adolescents are known to use the internet to experiment with their identities [19]. As the metaverse offers a new platform for social interaction on the web, one with additional features beyond the existing social media platforms, it will be both interesting and important to consider how people, and young people especially, interact with it while forming their identities.

## Discussion and Initial Recommendations

In this brief and incomplete discussion, we have compared and contrasted the metaverse with existing technologies. It is our view that the existing benefits and harms that these technologies provide are likely to continue into the age of the metaverse, should it materialize. However, we also believe that there are qualitative differences between this novel technology and existing technologies that provide us with new questions to ask, new potential harms to anticipate and mitigate, and opportunities for improving mental health services and developing new therapies and measures.

Research and policy directions will depend on our ability to predict the direction that the metaverse will take. We posit that this, in turn, depends on the answer to the following question: *cui bono* (who benefits)? Relevant to the public health perspective of this technology is this need to understand how metaverse architects will profit from it. For example, as social media networks are free to access, their business model focuses on driving user engagement to increase advertising revenue [33]. This is concerning, as the passive use of social media (eg, scrolling through news or content as presented by the site in its effort to drive engagement) was linked to reduced well-being in some studies, whereas active use (eg, messaging friends) was not [34]; although, as discussed previously, this is not a consistent finding in the literature. Similarly, the business models underlying the metaverse will drive how it is built and the behaviors it encourages, which in turn will drive its mental health effects. We believe that it is critical for researchers to consider this when designing studies and hypotheses as we cannot rely on companies to share data on worrying trends. Indeed, in a recent commentary on the private regulation of neurotechnology [35], the authors note that paying out settlements when harm is done is something that has simply become part of the “cost of doing business.”

Hence, we as users must question how we will be protected from the potential harms of the metaverse and who will be responsible for regulating it. The argument for the regulation of technologies related to social media and, as such, social media companies is easier to make in light of recent events described previously. However, to date, we have not been able to resolve the question of who should regulate these platforms and if regulation were to occur, how to balance the need to protect the public with the right to free expression and participation in public spaces.

Governments often lack the technical knowledge required to create and enforce regulations of technologies and can move slowly, and given the rising need for mental health services, there is an argument for making sure that the benefits of the metaverse to mental health can be realized quickly. In addition, governments are susceptible to lobbying, bribery, and collusion. By contrast, if companies are given free rein to self-regulate, the situation we currently face where social media companies put their bottom line above user safety will simply repeat in the metaverse. If we consider the past to be a good predictor of the future, then, in our opinion, early involvement of government regulation (in jurisdictions where there is adequate protection for freedom of speech) will be necessary to avoid a repeat of the status quo.

Then there is the question of what kind of regulation these platforms should be subject to. Although a full treatment of this subject is beyond the scope of this paper, we make some recommendations based on our view of the current and likely future situation for consideration by relevant authorities. First, we differentiate between those applications that are designed as treatments or diagnostic tools that are implemented in the metaverse and the metaverse more broadly. In the former, narrow case, our recommendations are outlined in Textbox 1.

This would be in line with existing regulations regarding devices designed to diagnose or treat illness in the United States, Europe, and Canada [36-38], and the argument for equivalent regulatory practices has been made for similar novel technologies such as artificial intelligence–powered medical products [39,40]. As such, the recommendation is not meant to change the status quo but rather to serve as a reminder that the creators of novel technologies continue to have responsibilities laid out in current regulations and that regulators, in turn, must not only be vigilant with respect to the unregulated deployment of new technologies but also, as the Food and Drug Administration has done in their new draft guidance on artificial intelligence devices [39], be innovative to ensure that their regulations and guidance evolve to best address novel technologies. In addition, we believe that care must be taken to streamline regulatory processes and provide templates and materials that can be used by smaller firms and start-ups, so that their entry into the market is not blocked by the cost of regulatory compliance. This is relevant not just from an economic standpoint because if smaller, newer firms cannot enter the market, then it will necessarily be dominated by existing social media giants, reducing the chance that the development of the metaverse could proceed in a different manner to the recent development of social media.

With respect to the metaverse more generally, we make the recommendations outlined in Textbox 2.

**Recommendations regarding applications designed as treatments or diagnostic tools.**

**Classification as medical devices**

- Any application developed in the metaverse ecosystem designed specifically as a treatment or diagnostic tool for mental health should come under regulations governing medical devices and require appropriate validation and evidence of safety and utility.

**Recommendations regarding the metaverse more broadly.**

**Increased transparency and control for users**

- Companies should be required to make available user-friendly suites of data to consumers describing how long they spend in the metaverse, what activities they engage in, how their data are being used, what kinds of targeted elements (eg, advertisements) they interact with, and how these were targeted. Users should be able to easily set limits on time, targeted marketing, and uses of their data. Although there is some evidence that most users do currently use tools such as advanced privacy settings on social media [41], their overall effectiveness in enhancing privacy and well-being is understudied and often questioned (see the study by Mondal et al [42], who discuss this in the case of Twitter). As such, this recommendation is included as a “baseline” of sorts that we believe, on ethical grounds, to be necessary though likely not sufficient for the mitigation of potential harms.

**Active moderation**

- We argue that from the start, companies must put in place measures to limit sexual and other forms of harassment and remove offending users from the platform. Users must be given tools to limit harassment, for example, being able to exclude other users from their personal space. In addition, effective reporting mechanisms should be put in place. One case study of the deplatforming (a form of moderation where controversial figures are removed from social media platforms) of 3 well-known influencers demonstrated that, at least on the platforms from which they were removed, the overall toxicity and activity of their supporters dropped after deplatforming [43], although some supporters may simply move to other less-moderated platforms, and the effectiveness of different moderation approaches—only some of which focus on the experience of individuals being subjected to harassment—remains an area of active research [44]. There are, of course, concerns regarding the open exchange of ideas and freedom of speech that must also be addressed when it comes to moderation. Although this is perhaps less of an issue in the case of clear interpersonal harassment, the many forms that harassment and intimidation can take and their ability to be directed to both groups and individuals will require careful legal and ethical analysis that is outside the scope of this paper. As such, we conclude that the precise form that active moderation should take in the metaverse is a question that will require experimentation, ethical debate, and research; therefore, a key element of this recommendation is that active and both prospective and retrospective analysis of the effectiveness and ethics of different moderation techniques be undertaken as the metaverse is implemented. During this research, care will be needed to differentiate between moderation that targets the macrolevel experience (ie, moderating popular figures that can influence the tone of discourse in the metaverse) and microlevel experiences of harassment between individual users [44]. This prospective approach to shaping moderation practices would be in contrast to what is arguably the more reactive approach to moderation taken in social media in recent years.

**Compulsory after-market research**

- Companies should be required to collect data, in aggregate form using a format mandated by regulators, on user mental health and share this with relevant authorities and the research community, very similar to how pharmaceutical companies are required to complete after-market studies.

**Compulsory beta testing and data sharing**

- Companies should be required to extensively beta test the metaverse and collect data on health outcomes in a prespecified manner by regulators and in representative populations, and this beta testing should be required for major application updates. This information should be submitted to regulators who may then take appropriate actions. This information should also be made public and available to researchers. This and the previous recommendation are based on the consistent findings in the literature, discussed previously [7,8], that the quality of and access to data has been a challenge in social media research. As such, creating programs for structured beta testing as well as postmarketing research, the data for which are intended to be generated in a format meant for sharing with researchers and regulators, should help accelerate the pace and increase the quality of metaverse-related research. It would also help avoid situations such as those described previously in connection with social media, where evidence of potential harms experiences significant delays before being made public.

**Taking privacy protection seriously**

- Social media companies and data aggregators are currently exempt from health data privacy regulations such as the Health Insurance Portability and Accountability Act (HIPAA), as they are not considered creators or custodians of health care data. However, the data they aggregate can contain detailed information about an individual, and the behavioral measures available in the metaverse may exacerbate this situation [45]. Steps could be taken to bring any company that controls or collects data that can be used to generate a profile of a person’s health status under relevant regulations such as HIPAA. However, defining which data come under this definition will be a challenging exercise, and as noted previously, care must be taken to streamline requirements such that smaller, innovative firms are not frozen out of the market to the benefit of existing major players in the space.

It should be noted that, to properly regulate the metaverse, a clear operationalization of the metaverse is necessary to allow regulators to know what to regulate and in which contexts. If an application, regardless of its precise implementation, is generated with a clearly medical purpose and makes medical claims, it would be relatively easy to argue that it should be regulated as a medical device (as we do in Textbox 1). However, the precise definition of what is part of the metaverse becomes more important when the application in question is not clearly medical in nature. The description of the metaverse as a virtual environment where people work, play, interact, and receive services using VR is a starting point for a definition but one that will need to evolve as the metaverse takes shape. We posit that the question for regulators will then become *which* elements of this space and its construction may have health impacts that require regulatory oversight. We believe that this may be, in essence, an empirical question, one that could be answered by the compulsory after-market research and data sharing we described in Textbox 2. Once sufficient data have been collected and scrutinized by regulators, researchers, and the public, it may become easier to see which nonmedical elements of the metaverse are most deserving of health-related regulation. We argue that this would have been a helpful approach in the original deployment of social media, helping to maximize benefits and minimize harms; there is now an opportunity to approach the nascent metaverse in this manner.

In terms of the limitations of this work, one area of technology that we have not addressed is digital gaming, an area with a rich research literature. This is because, to date, in the opinion of the authors, the metaverse has been focused on the union of digital work and social media in a VR space, and it is not yet clear to us what transformative impact they will have on the experience of digital gaming. However, such impacts may occur and should be the subject of future research.

## Conclusions

The challenges and potential uses of the metaverse and similar VR communities merit meaningful discussion to maximize the benefits and limit harm. In summary, our viewpoint is as follows: the metaverse is a tool that can facilitate mental health care and treatment and that has vast potential to provide innovative approaches to the measurement, detection, and treatment of mental illness, so long as it is used appropriately. We can best ensure that this occurs through research, proper prospective data collection, and proper use of the data collected. Governments and other regulators should become involved now, before it is too late, to protect the metaverse from becoming just another commercial space devoid of standards to protect users. This is particularly relevant to mental health after the COVID-19 pandemic, where virtual care is already a standard of care, as the metaverse is poised to become the next platform for virtual mental health care delivery.

## Abbreviations

VR: virtual reality
